# A Systematic Review of the Current Hepatitis B Viral Infection and Hepatocellular Carcinoma Situation in Mediterranean Countries

**DOI:** 10.1155/2020/7027169

**Published:** 2020-06-10

**Authors:** Salma Madihi, Hashim Syed, Fatiha Lazar, Abdelmajid Zyad, Abdelouaheb Benani

**Affiliations:** ^1^Molecular Biology Laboratory, Pasteur Institute of Morocco, 1 Place Louis Pasteur, Casablanca 20360, Morocco; ^2^Team of Experimental Oncology and Natural Substances, Cellular and Molecular Immunopharmacology, Sultan Moulay Slimane University Faculty of Sciences and Technologies, Beni Mellal, Morocco

## Abstract

Viral hepatitis B is a global public health problem affecting nearly two billion subjects; 3.3% of whom are from the WHO (World Health Organization) Eastern Mediterranean Region (EMRO). It induces both acute and chronic hepatic disorders with subsequent liver cirrhosis and hepatocellular carcinoma (HCC) in a considerable percentage of patients based on the age of exposure. In this review, hepatitis B virus (HBV) and HCC prevalence, distribution and prevalence of different genotypes, and male/female infection frequencies in relation to the vaccination status in the Mediterranean countries were reported. *Study Design*. This systematic review describes the prevalence of hepatitis B infection, genotype distribution of hepatitis B virus, and prevalence and incidence of hepatocellular carcinoma in Mediterranean countries belonging to three different continents: Southern Europe (Spain, France, Italy, Croatia, and Greece), North Africa (Morocco, Algeria, Tunisia, Libya, and Egypt), and the Near East region (Syria, Lebanon, Turkey, Israel, and Palestine). We tried to collect new data from electronic databases: PubMed, ScienceDirect, ResearchGate, Google Scholar, and public health reports between 1980 and 2019. For each publication, we recorded reference, publication year, study characteristics (date, locations, sample size, and study population), and participant characteristics (population group, year, age, and sex). No language limitation was imposed, and articles or reports from non-peer-reviewed sources were not considered for this analysis. The main keywords were HBV prevalence, hepatitis B infection, HBV genotype, and HCC. *Inclusion and Exclusion Criteria*. Healthy population-based studies included the following sample populations: (i) voluntary blood donors, (ii) pregnant women, (iii) community studies, (iv) hemodialysis patients, (v) hospitalized patients, (vi) healthcare workers, (vii) sex workers, (viii) drug abusers, and (ix) prisoners. We excluded studies from the following special groups who were assumed to be at a special high risk: patients from sexually transmitted disease clinics and thalassemia clinics and professional or paid blood donors.

## 1. Introduction

Viral hepatitis B is a prevalent infection caused by the hepatitis B virus (HBV) and is the leading cause of acute and chronic liver diseases worldwide. The WHO (World Health Organization) estimated that the number of people exposed to this virus to be roughly 2 billion; 240 million of whom are chronic carriers [1]. Additionally, the WHO estimates the number of HBV-related deaths from liver cirrhosis and HCC is 1.34 million deaths per year [2]. The persistence of the disease in humans is due to virus's complex life cycle and its ability to utilize few protein products in a multifunctional way to persist and escape immune detection and elimination [3].

HBV is the smallest enveloped dsDNA animal that belongs to the *Hepadnaviridae* family, and its DNA genome is an incomplete double strand of 3.2 kb [4] organized into four open reading frames that overlap and code for HBc capsid, HB surface protein (envelope), reverse transcriptase, and nonstructural protein X (HBx) [5], which is known for its oncogenic properties and believed to induce hepatocellular carcinoma (HCC). This protein makes chronic viral hepatitis B one of the most important etiologies leading to the development of primary liver cancer [6] as 55% of patients showing protein X expression developed HCC later on [7].

### 1.1. HBsAg Prevalence

HBV infection is widespread worldwide and unevenly distributed, with three resultant geographic categories to describe endemicity: (i) areas of high endemicity (>8%) characterizing mainly developing countries (Sub-Saharan Africa, South East, and Far East Asia), (ii) areas of intermediate endemicity (2-7%) which cover the Mediterranean, Eastern Europe, and Latin America, and (iii) areas of low endemicity (<2%) represented by Western Europe, North America, and Japan [8]. In the WHO Eastern Mediterranean Region, an estimated 3.3% of the general population is infected [2]. The modes of transmission vary slightly from one country to another, due to the differences in the blood transfusion safety protocols and preventive measures implemented by the governments. Meanwhile, HBV transmission is predominantly horizontal, resulting from the exposure of abraded skin, cuts, minor open wounds, or mucosal surfaces to blood or body fluids containing HBV from the afflicted subjects [9].  Table 1 represents recent data about HBsAg prevalence among different study populations in all the Mediterranean countries reported in this review.

### 1.2. HBV Genotypes and Subgenotypes

Regarding HBV genotypes; ten types (A to J); four serotypes, adw, adr, ayw, and ayr; and many subgenotypes have been described with distinct geographic distributions and several HBV mutants, including precore/core promoter mutations and pre-S/S deletion mutations, based on the sequence divergence of the HBV genome, with a minimum of 8% nucleotide sequence variability [10]. HBV genotypes and subgenotypes among patients belonging to Mediterranean countries are presented in Table 2. HBV genotype-specific pathogenesis may contribute to heterogeneous clinical outcomes in chronic hepatitis B patients across the world, increasing the risk of cirrhosis and HCC development [10].

### 1.3. HCC

Hepatocellular carcinoma is the main primary malignant tumor of the liver. Globally, it is the fifth most common cancer in men and the seventh among women cancer in terms of incidence with more than 700,000 new cases being diagnosed each year, and the third leading cause of cancer death with chronic development and progression [11] and over 600,000 deaths globally per year, accounting for 9.2% of all new global cancer cases (7.9% in men vs. 3.7% in women) [11]. It is a complex process due to several factors: inflammation, DNA damage, epigenetic changes, senescence and telomerase reactivation, chromosomal instability and early neoangiogenesis, and mostly due to hepatitis B and C viruses. HBV contributes to HCC development in more than 50% of cases [11], and it is known to be a group 1 human carcinogen and a highly oncogenic agent [12]. One of the most important mechanisms of HBV's direct prooncogenic role is its ability to integrate into the genome of the infected host hepatocytes. Integrated viral DNA has been found in 85–90% of HBV-related HCCs. HCC can be developed in HBV patients without any previous history of either liver lesion or cirrhosis in 20% of all HBV-related HCC cases. However, cirrhosis is an important predisposing factor to HCC also in cases with HBV infection [13]. Regarding HBV variability and HCC, Asian studies reported that HBV genotype C is associated with more aggressive liver disease towards cirrhosis and the development of HCC, compared with genotype B, whereas in Western Europe and North America, genotype D is more associated with a higher incidence of HCC than genotype A [11]. We reported the recent data about HCC incidence, male/female ratio, and etiologies in each Mediterranean country in Table 3.

### 1.4. Prevention

To prevent hepatitis B and subsequent HCC development, the vaccine is the main mainstay. It has been available since the 1980s, and it is the first vaccine to protect against cancer by reducing the incidence of HCC in highly endemic areas by 75%. In 1991, WHO recommended the incorporation of this vaccine into the Expanded Program on Immunization (EPI). The immunization program, according to WHO, targets all infants, preferably within 24 hours of birth [14] and anyone belonging to high-risk groups: people with frequent need for blood transfusions, dialysis patients, and organ transplant recipients, inmates, injecting drug users, sexual partners of infected persons and people with multiple sexual partners, people sharing a home with chronically infected people, and health workers and travelers wanting to travel to a high-endemic area [15]. There is also a postexposure vaccination concerning cases after an accident of exposure to blood or after unprotected sexual intercourse in nonimmunized subjects, newborns of mothers carrying HBsAg, in prevention of hepatitis B relapses after liver transplantation and in postexposure in personnel not responding to vaccination. The postexposure vaccination is based on the administration of immunoglobulins as quickly as possible, preferably within 24 to 72 hours, at most one week after exposure [15]. Vaccination dates of all Mediterranean countries are represented in Table 1.

### 1.5. Morocco

Before the introduction of the hepatitis B vaccination into the immunization program, the WHO concluded that Morocco has an intermediate prevalence of hepatitis B [16], an infection that remains a public health problem in the country. Morocco is a country that has adopted the strategy of vaccinating infants from birth since the 1999s, and since 1987, HBsAg screening in blood donors has become mandatory, with a systematic clinical examination of the donor by a doctor since 2004 [17]. To determine transmission modes in Morocco, Bennani et al. published in 2011 results of a large screening of HBsAg and reported that HBV is parenterally and sexually transmitted in the country. The study revealed that HBV prevalence was about 1.79% of the general population studied [18]. Another epidemiological study between December 2005 and June 2011 of 23,578 apparently healthy Moroccan subjects collected randomly from eleven major Moroccan regions revealed a 1.81% prevalence of HBsAg. In all age groups, the HBV positivity was significantly higher among males than females, especially among ones aged between 30 and 49 years (2.4%) [19]. This result could reclassify Morocco as a low-endemic area for HBV. The same study reported that, between January 1, 2008 and December 31, 2010, only 0.96% of the volunteer Moroccan blood donors who donate blood at the Blood Transfusion Center in Casablanca had HBV infection, corroborating Baha et al.'s estimates of low donor prevalence [19]. Another ten-year retrospective study screened the seroprevalence of HBV in 19,801 volunteer healthy blood donors and showed that only 0.8% were HBV carriers. Interestingly, it was also confirmed that seropositivity of HBV decreased from 1991 to 2010 [20]. On the other hand, scarce data was reported about the HBV prevalence in hemodialysis Moroccan patients. A transverse study screened 67 chronic hemodialysis (CHD) subjects, 31 males and 36 females aged between 25 and 88 years, showed that only four subjects (6%) were tested positive for HBsAg, and two of them have confirmed infected prior to their admission in the CHD [21]. Recently in Morocco, over a period of three months (February, March, and April 2019), a prospective, observational single-center study on pregnant women in Agadir was conducted on pregnant 483 women with an average age of 29.9 ± 6.8 years. The study reported that HBsAg was found in 1.2% of subjects [22].

Accordingly, Morocco remains classified in the zone with low endemicity. In Morocco, data on HBV genotypes was collected for the first time in 2007. After genotyping was performed on 40 HBV-positive individuals, 39 were determined to be infected by genotype D (97.5%) and a single patient with genotype A (2.5%) [16]. Similarly, two studies in 2008 and 2011 revealed that 100% patients with chronic hepatitis B were found to possess genotype D [18, 23]. In 2012, 200/221 patients with chronic hepatitis B were found to have D genotype (90.45%), 13/221 A genotype (5.9%), 1/221 E genotype (0.5%), and 7/221 mixed genotypes (3.17%) (5 A/D and 2 D/F). The dominant subgenotype was D7 (63.3%) followed by D1 (32.7%) while each of D4 and D5 showed 2%. Meanwhile, A2 was the only detected subgenotype [24]. Despite the predominance of primarily D and A genotypes, the modes of contamination are diverse; vertical, intrafamilial, sexual, or parenteral modes of transmission are possible [25].

In Morocco, HCC represents 5.9% of the total tumor burden [26] and its annual incidence is estimated to less than 4/100,000 people/year, according to GLOBOCAN 2008 [27]. A study from 1st January 2001 to 31st December 2015 was elaborated on 440 cases of HCC (62.5% of males and 37.5% of females) ranging from 21 years to 89 years, with a sex ratio male/female of 1.7. Cirrhosis liver was detected in 144 patients (32.7%) with indeterminate cause in 61 (13.8%) of them. 307 (69.7%) patients were tested positive for hepatitis C virus (HCV) while HBV was present in 67 patients (15.2%) and 4 patients (0.9%) were considered chronic alcoholics [26]. According to new data about HCC etiologies, HBV contributes in 31% of HCC cases, while HCV, alcohol, and other factors contribute in 36, 14, and 19%, to HCC development, respectively [28].

### 1.6. Algeria

In Algeria, the Algerian Ministry of Health introduced vaccination against B virus in the vaccination calendar in September 2002, routine screening of HBV and HCV in blood and organ donors in May 1998, and also in people exposed to risk in April 2000 [29]. Based on the WHO standards, Algeria is considered to have intermediate prevalence with estimates of 2-7%. In 1995, a study was conducted to assess HBsAg and anti-HBc as serological markers in 1112 apparently healthy blood donors and 715 pregnant women in different regions in Algeria. HBsAg was detected in 3.6% of blood donors and 1.6% of pregnant women [30]. In 1998, a national survey established the prevalence of HBsAg to be 2.15% [31]. In 2011, 576 patients (60% males and 40% females) on CHD with a mean age of 57.5 years were screened in eight dialysis centers in Constantine. Results showed that HBV was positive in 3.2% [32]. In 2013, in order to test new chronic infection cases, 2946 samples were collected from 41 administrative regions covering 92% of the population in Algeria. The study reported that, among the 2946 samples tested for HBV load measurement, 1876 subjects were newly diagnosed chronic HBV infections and detected positive for HBsAg (54% were males). The mean age of patients was 36.8 ± 14.2 years [33]. These results suggested an intermediate HBV prevalence in the general population in Algeria; however, a low prevalence in other demographics such as pregnant women was reported.

Concerning HBV genotypes in Algeria, it has been revealed that among 75 chronic HBV carriers in the northeastern region of the country, genotype D was found to be the predominant HBV type (93%) followed by A (5%) with a single patient having a genotype E [34]. Recently, S1 sequencing of HBV from 119 patients revealed the presence of genotypes D (86.5%) and A2 (11.76%). Phylogenetic analysis of the D genotype strains clustered them into D7, D3, D1, and D2 in 43.5%, 24.75%, 16.8%, and 14.85%, respectively [35].

In Algeria, the incidence of HCC is between 4 and 7.9/100,000 people/year, according to GLOBOCAN 2008 [27].

### 1.7. Tunisia

Prior to the introduction of the universal vaccine program in Tunisia in 1995, hepatitis B prevalence was evaluated at 5.5% in 1990 [36]. Then, during 1996, a seroepidemiological population-based cross-sectional study of 9486 volunteers in two governorates, Beja in the north (*n* = 2223) and Tataouine in the south (*n* = 7235), was conducted. The overall prevalence of HBsAg in the two regions was 4.2% and 5.6%, respectively. The HBV-positive group was divided into three subgroups: anti-HBc-positives, HBsAg-positive (tested for the first time), and HBsAg chronic carriers, for whom the HBsAg remained positive during the second sampling, 3 years after the date of the first sample. The study revealed that the overall prevalence of HBsAg and chronic carriage was 5.3% and 2.9%, respectively. The male-to-female ratio was 1.06 : 1 for HBsAg subjects and 1.09 : 1 for chronic carriers, and the prevalence in males was significantly higher compared to females: 6.4% vs. 4.5% for HBsAg. The mean age of HBV-tested subjects was 26.3 ± 20.7 years [37]. In 2008, 2303 Tunisian pregnant women were tested for HBsAg among whom 4% were positive [38]. Later, a retrospective study of all blood donors at the Military Center of Blood Transfusion was carried using 198,157 available donor samples; 95% of which were men, aged between 20 and 25 years. The study reported that the prevalence of HBV among blood donors was decreased from 3.54% in 2000 to 0.8% in 2011 [36]. Concerning hemodialysis (HD) patients in Tunisia, a recent study by Mhalla et al. in 2018 reported results of a cross-sectional study between 2012 and 2014 showing an evidence of the presence of 5.5% HBsAg positive among a total of 109 HD patients (75 males and 34 females) tested for HBsAg and HBV DNA and ages ranged from 21 to 81 years [39].

The country is mostly characterized by genotype D. In 2006, 79 patients have chronic HBV infection and reported a predominance of genotype D (80%, *n* = 66) with the HBV-D7 is the dominant subgenotype followed by genotype A (9%, *n* = 7) and genotype E (8%, *n* = 6) [40]. In 2007, the predominance of genotype D via another similar study on 164 patients (84.75%) was confirmed. Rarely detection of genotypes A (0.6%), B (0.6%), and C (1.82%) and 20 mixed genotypes (12.2%) in the northern part of the country was also reported [41]. Both genotypes D and A were also detected in another study in the central-east Tunisia upon genotyping HBV strains from a total of 217 HBsAg-positive patients: genotype D 96% and genotype A 4%.

Phylogenetic analysis revealed 55% of strains belonging to subgenotypes D1, followed by D7 (41%) and only one strain with D3 subgenotype (3%) [42].

In Tunisia, chronic hepatitis B and C account for more than 75% of the etiologies of the HCC. According to GLOBOCAN 2012, the country is characterized by a low incidence of HCC estimated at 1.49 new cases/100,000 inhabitants [43] and is responsible for 1.1% of cancer deaths [26]. Recently, results of a retrospective study carried out from January 2002 to December 2017 including all HCC complicated post viral B (37%) or C (63%) cirrhosis hospitalized patients were published. The study included a total of 84 cases of HCC: 53 patients with HCV-HCC and 31 patients with HBV-HCC. Patients with HBV-related HCC, aged between 51 and 90 years, revealed that the male/female ratio was 9.33 [43]. Recent data showed that HBV, HCV, alcohol, and other factors contribute to HCC in 20, 44, 18, and 18%, respectively [28]. To overcome HBV and HCC problems in health institution, Tunisia provides vaccination to health professionals [44].

### 1.8. Libya

In Libya, hepatitis B vaccination was added to the EPI in 1993 [45]. In addition, Libya is a country that provides voluntary vaccination to people in high-risk groups for free since 1997 [44]. Both prenatal and horizontal transmissions were found to be important for transmission of HBV in the country. Until 2008, the estimated number of chronic HBsAg carriers in Libya was 120,000-150,000 individuals [46]. In 2008, a nationwide cross-sectional study was carried out on 65,761 individuals and showed that the prevalence of HBV varied based on the locality, age, and sex. The overall prevalence of HBV was 2.2%, and the affected male-to-female ratio was 1.4 : 1. The prevalence of HBsAg was 0.8-0.9% below the age of 10 years and slightly higher in individuals above 10 at 2.3–2.7% [47]. Concerning healthcare workers (HCWs) in Libya, between 28 February and 28 December 2011, a cross-sectional study was conducted on 2705 employees. Of age, they ranged from 17 to 74 years and the majority were females (60.1%). Results showed a prevalence of HBsAg estimated at 1.1% [45]. Additionally, in 2013, the prevalence of HBsAg was consistent with the 2008 report, 2.2%, while the risk factors for HBV infection in the country were family exposure and contact with HBV [48]. A study from 2008 to 2015 in the four different regions of Northeast Libya revealed a low prevalence of HBsAg among healthy blood donors; as among 78,987 subjects, only 0.21% were HBsAg positive [49]. A study between May 2009 and October 2010 on 2382 adult patients receiving maintenance HD in 39 Libyan dialysis centers reported a prevalence rate of 2.6% for HBV infection and mentioned that 58% of participants were males [50].

To determine the circulating HBV genotypes in the country, in 2012, 121 HBV-infected Libyan patients, 79 males and 42 females, aged 15-66 years, were genotyped. Results of the study revealed that genotype D was the most prevalent (90%) while both genotypes A and E were found in a single isolate each (1.7%). For 4 remaining samples, the authors suggested that the genotypes were mixed or recombinant D and E, representing 6.7% of genotyped isolates. The study included the precore region, where Salem et al. found that all 39 isolates investigated were genotype D and showed mixed wild type (G1896) and precore mutant (G1896A) [51].

In Libya, according to GLOBOCAN 2008, HCC incidence is estimated at 8-11.9/100,000 people/year [27]. Recent data showed that HBV is responsible for 33% of HCC development while HCV, alcohol, and other factors as aflatoxin B exposure are responsible for 34, 15, and 18%, respectively [28].

### 1.9. Egypt

In Egypt, the HBV vaccination program was applied in 1992 in order to reduce the prevalence of HBsAg in the country, confirmed by many studies to be moderately endemic with about 4% of the population having evidence of chronic HBV infection [52]. As an example, a meta-analysis between 1980 and 2007 reported that the prevalence for HBV in Egypt was 6.7% among the general population and 25.9% among HCC-infected persons. HBV was also found to be higher in the southern part of the country than the northern part, 11.7% and 4.6%, respectively. HBsAg was detected in 4% of pregnant women [53]. Concerning blood donors in Egypt, a study screened 55,922 potentially healthy asymptomatic blood donors with mean age of 30.98 ± 8.6 years; all of them were volunteers and about 94% were males. The seroprevalence of HBV infection was determined as 1.3%, and the authors mentioned a decline in seroprevalence from 2.3% to 0.9% among the general population in 2009 [54]. Additionally, in 2015, the Egypt Health Issues Survey conducted a cross-sectional analysis of men and women aged 15 to 59 and reported that, among the general population, 15,777 samples, 1.4% were found positive for HBV. The overall prevalence rate for HBV among males was 1.9%, compared to 1.1% for females [55]. Recently in 2017, a cross-sectional study was carried out on 641 patients who agreed to give informed consent to participate. The patients had end-stage renal disease on regular HD with a mean age of 53.18 ± 13.26 years. 2% of them were HBV positive with 1.6 : 1 male-to-female ratio [56]. Between June 2014 and April 2015, a cross-sectional study was conducted among 564 workers in governmental (*n* = 461) and nongovernmental (*n* = 103) hospitals in Tanta City, Egypt. HCWs showed high exposure to HBV (24.5%); however, active infection was recorded only in 1.4% among the examined 564 subjects [57].

To explore HBV genotypes in Egypt, in 2003, 105 HBsAg-positive serum samples collected from blood donors and chronic HBV patients in North Egypt were found to be related to genotype D. Furthermore, phylogenetic analysis based on the complete genome sequences revealed that genotype D in Egypt was close to that in Mediterranean countries with a high degree of nucleotide homology (97.3%) [58]. Among patients with acute hepatitis B (AHB), patients with chronic active hepatitis (CAH), and patients with HCC in Egypt, a study in 2011 on 140 patients showed that genotype D constituted 87% of the total infections. The other 13% were mixed infections of D/F, only encountered in AHB patients [59]. Additional study investigating HBV genotypes in Egypt was performed between June 2014 and April 2015; genotype E was detected in 50% of the samples while genotype D was found in 21.43% and coinfection with E and D was reported in the remaining patients (28.57%) [57]. Recently, in 2017, genotype D (subgenotype D1) was detected in 38 HBsAg-positive patients (20 females and 18 males aged 21-54 years) from Mansoura City and its surrounding villages [60]. In cancer pediatric patients, HBV genotypes were determined in 22 patients who had AHB and in 48 patients with CAH. Genotypes D and B constituted 37.1% and 25.7%, respectively, in addition of mixed infections of 15.7% among the studied group especially mixed A/D genotype infections [61].

Regarding HCC situation in Egypt, the cancer constitutes a public health problem and it is responsible for 33.63% and 13.54% of all cancers in males and females, respectively. Its incidence was estimated to be more than 20/100,000 people/year, according to GLOBOCAN 2008 [27]. A study from January 2011 till the end of 2016 on 300 HCC cases reported that 81.5% were males and 18.5% females and 53% of the studied HCC patients were younger than 60 years old [62]. New data showed that HCC etiologies are different: HBV (13%), HCV (63%), alcohol (12%), and other factors as aflatoxin B (12%) [28]. HCC is strongly linked to HCV in Egypt as the country is known to have one of the highest prevalences of patients with HCV worldwide [62].

### 1.10. Syria

In Syria, hepatitis B vaccine was added to the national vaccination program in 1993.

However, there are no estimates of children who received the 1st dose of hepatitis B vaccine within 24 h of birth, as home delivery is still common in the country [63]. Earlier in 2002, it has been reported in Syria that HBsAg positivity was 5.3% and 10.8% in drug users and sex workers, respectively [63]. In 2004, a large survey on a random cluster sample with 528 clusters and 3168 individuals revealed the presence of HBsAg in 5.6% of subjects. There was a clear regional variation in the prevalence of hepatitis B, and a higher prevalence was mainly reported in two governorates in the northern (Aleppo) and eastern parts of the country (Hassakeh), where the seroprevalences for hepatitis B were 10.5% and 10.6%, respectively [63]. Between October 2012 and December 2013, a study on 159 multitransfused patients was established (88 males and 71 females). Among the 159 patients, 1 (0.63%) was positive for HBsAg and 4 (2.52%) were positive for anti-HBc [64]. A recent retrospective review was conducted between April 2014 and December 2015 on 171 Syrian refugee children (51% were females) aged between 0 and 18 years. 140 patients of 171 were scanned for HBV, and 6 of the 140 patients (4.2%) were HBsAg and anti-HBc total positive and anti-HBs negative [65].

Between January 2012 and January 2018, a total of 11,015 Syrian pregnant women with a mean age of 25 ± 6.02 years were examined retrospectively and showed a rate of HBsAg seropositivity of 1.1% [66]. HBV genotypes were tested on a total of 220 patients from nine medical centers in Syria in 2008. Patients were aged between 14 and 85 years, and 181 (82%) patients were males. Genotype D was the predominant type (213 of 220, 97%) while genotypes A, C, F, and D/H were rarely detected [67]. Between August 2008 and April 2010, fifty Syrian hepatitis B patients with high viral loads (more than 6 log) were enrolled in the study. Of them, 52% were treatment-naïve and 48% treated. As a result, all patients had genotype D [68]. In Syria, many factors are responsible for HCC development. New data revealed that HBV, HCV, alcohol, and other factors contribute to HCC in 32, 34, 14, and 19%, respectively [28].

### 1.11. Lebanon

In Lebanon, the current status of hepatitis B infection is not well known due to the lack of published studies on the subject. However, we tried to collect data about the different populations and summarize the current situation of the problem. Earlier in 1972, prevalence and incidence of hepatitis B were investigated in Lebanon and ranged between less than 2% and more than 3%. Then, after hepatitis B vaccination of newborns was included in the Lebanese system in 1998, prevalence decreased among the general population [69]. In 2007, the WHO estimated that hepatitis B prevalence ranges between 1.6% and 2.2% [69]. During the same period, between August 2007 and February 2008, a total of 580 male prisoners aged 16 and above were randomly selected from four prison blocks in Lebanon and tested for HBV markers. HBV was recorded among 2.4% of prisoners [70], a significantly higher seroprevalence compared to the general Lebanese population. In the same period, between August 2007 and July 2008, a biobehavioral surveillance study was carried out on 204 subjects: men who have sex with men (MSM) (*n* = 101) and female sex workers (FSW) (*n* = 103), aged 18 years and above. As a result, only 1 person was detected HBsAg positive in sex workers from both genders (0.99%) [71]. Later, a cross-sectional study conducted from January 2011 to December 2012 on 31,147 subjects from six Lebanese governorates revealed the presence of HBV in 542 subjects (1.74%) using a rapid test with a male-to-female ratio of 1.08 : 1. HBV exposure was higher in the South and Nabatiyeh (1.9%) in comparison to subjects from Beirut (0.73%) [69].

Lebanon, as all Mediterranean countries, is characterized by HBV genotype D. In this regard, a study of 61 HBV carrier blood donors from Lebanon was performed between July 2009 and January 2011. All HBsAg-positive plasma samples were from Lebanese male donors ranging between 18 and 60 years (median, 35 years). Genotype D was the only type detected (100%) (serotype ayw), with the majority of the strains (*n* = 35) found related to subgenotype D1 and few strains (*n* = 7) related to subgenotype D2 [72]. HCC in Lebanon is due, mostly, to HCV in 48%, HBV in 20%, alcohol in 15%, and other factors in 17% [28].

### 1.12. Palestine/Israel

Israel is a country that provides vaccination to health professionals, and the vaccine has been given since 1992, resulting in a significant reduction in morbidity [44]. In 2007, 246 patients (131 males and 115 females) from the four governmental HD centers of Palestine showed an overall prevalence of 8.1% of HBsAg [73]. Later, during the period from October to November 2014, 33 out of 868 (3.8%) HD patients in the West Bank hospitals in Palestine were positive for HBsAg and the prevalence ranged from 0.0% in Jericho and Qalqelia to 11.8% in Bethlehem [74]. Recently, a study showed that 868,714 people (22.6%) were exposed to HBV while 15,258 people were HBsAg positive (1.75%). The prevalence was higher in the Arabic population than in the Jewish one: 2.98% and 0.76%, respectively, and males represented 59.6% vs. 40.4% for females among the positive subjects [75]. In this region, two HBV genotypes, with different subgenotypes, are detected. A molecular analysis, by Shirazi et al., was performed between 2010 and 2015 on 58 patients positive for HDV RNA and 27 HBV-monoinfected patients. HBV genotype was determined in 33 samples: 6 HBV/HDV patients and 27 HBV-monoinfected patients. Results of the 6 HBV/HDV-infected patients revealed 66.7% of subgenotypes belonging to D2, 16.7% to D1, and 16.7% to D3. In contrast, of the 27 HBV-monoinfected patients, the distribution was as follows: D1 (59.3%), D2 (18.5%), D3 (11.1%), and A1, A2, and C2 (3.7%) each [75]. In 2014, 40 HBsAg-positive serum samples from Al-Makassed Islamic Charitable Hospital were subjected to HBV subgenotype analysis. The genotype D was prominent among Palestinian patients while genotype A was less commonly detected. D1 subgenotype was detected among Palestinians in 90% of cases; meanwhile, one (2.5%) sample belonged to the D3 subgenotype and three (7.5%) to the A2 subgenotype, respectively [76]. To determine HCC incidence in this area, a large national study from January 1st 2000 on all members without cirrhosis or cancer followed until death and disenrollment on January 2017. 1,129,969 subjects with a mean follow-up time of 15.15 years were included. Results revealed an overall incidence rate of diagnosed cirrhosis estimated at 1.85/10,000 persons/year and 0.75/10,000 persons/year for liver cancer. Authors concluded that the incidence reduced to 1.05 for cirrhosis and 0.58 for liver cancer after excluding patients with viral hepatitis or significant alcohol consumption (4.1%) [77]. In Israel, HBV is responsible of 20% of HCC cases, while HCV, alcohol, and other factors are responsible for 49, 15, and 17%, respectively [28].

### 1.13. France

In Europe, France was among the first countries to propose HBV vaccine to healthcare workers and high-risk populations, since 1982 [78], which makes the country characterized by a low endemicity estimated at 0.65% in the adult metropolitan population with an incidence of symptomatic AHB at 1/100,000 inhabitants [79]. Early between 1984 and 1998, a fifteen-year study was carried out on pregnant women and the overall prevalence of HBV was 0.65%. Low prevalence was observed in women of French origin (0.29%), 5.68% in women from French West Indies islands, 7.14% in women of foreign origin especially South East Asia, and 6.52% in women from Africa (Sub-Saharan) [80]. In the country, the HBV prevalence rate decreased between 1993 and 2000 by a factor of 2.5 for HBsAg in autologous blood donors and was estimated as 0.12% [81]. Another fifteen-year survey from July 2001 to December 2015 was performed, in which 16.5 million volunteer blood donors were screened for HBV, and reported that only 1583 subjects were found positive. This study revealed a great benefit related to HBV screening with a rate of 0.88 per million donations [82]. Concerning end-stage renal disease patients, 72,948 subjects were tested for HBV from January 2005 to December 2013. The prevalence of HBV was 0.84%, and 62.5% of HBsAg-positive patients were men. By age group, the HBV prevalence increased progressively until a maximum rate at 1.80% in the 4th decade and then regularly decreased [83]. In France, genotype G was initially reported in 2000 [84]. Later in 2005, a national multicenter retrospective cross-sectional study was established to correlate the presence of extra hepatic manifestations with HBV genotypes in patients with chronic HBV infection. HBV genotypes were determined in 190 patients HBsAg positive for at least 6 months and were mainly males (77%). The detected HBV genotypes included D (29%), A (24%), C (11%), and E (10%) [85]. Another multicenter retrospective study on 262 patients with chronic HBV infection was conducted in 2005 which revealed the presence of genotypes: D (27%), A (24%), E (13%), C (12%), and B (7%). Mixed genotypes were detected in 16% of the cases [86].

HCC in France accounts for approximately 8000 deaths per year, and the prognosis is one of the poorest compared to all cancers [87]. In this regard, a retrospective analysis of French healthcare databases was elaborated between 2009 and 2012 on 16,641 included patients diagnosed with an incident HCC. 14,060 incident cases were alcohol-related HCC, and 2581 were HCV-related HCC. Males represented 88.8% and 62%, and the mean age was 66.9 ± 9.3 and 68.1 ± 12.9 years in the alcohol-related HCC and HCV-related HCC groups, respectively.

Results showed that alcohol-related HCC was more frequent than HCV-related HCC (29.37/100,000 vs. 5.39/100,000 adults/year), with an important particularity of alcohol-related HCC in the north and west parts [87]. Recent results of a study conducted from October 2010 to April 2016 showed that among 652 patients with alcoholic cirrhosis included in 22 French and Belgian centers, HCC was diagnosed in 43 patients. The incidence of HCC was 2.9/100 patients/year and one- and two-year cumulative incidences of 1.8% and 5.2%, respectively [88].

### 1.14. Spain

In Spain, until the early 1990s, selected hepatitis B vaccination strategy was adopted for high-risk groups exclusively [89]. It is a country with an intermediate HBsAg prevalence (2-8%), defined by the WHO. Studies from 1996 to 2013 in different regions in Spain were elaborated on juvenile and adults randomly chosen in Catalonia, randomized adult population from Gijón, people chosen at random out in Catalonian countries, and pregnant women and general population of an urban public health area in Castilla and Leon. Prevalences were 0.7%, 1.2%, 1.69%, 0.8%, and <0.27%, respectively, suggesting a considerable reduction [90]. In 2005, a longitudinal observational study of 381 pregnant women was carried out and showed that the prevalence was around 0.8% [91]. Then, from October 2007 to February 2010, 5017 volunteers and showed that the prevalence of HBV-related serum markers in a healthy employed population of Murcia and Madrid is lower than described in prior papers referring to 0.7% for HBV [92]. Later, between January 2013 and 2014, 1.03% of the 15,645 Spanish HD patients from 215 centers showed chronic HBV infection [93].

In 2002, 258 Spanish western patients with chronic hepatitis B were found to be infected with many different HBV genotypes, showing the prevalence of genotypes A, D, and F as 52%, 35%, and 7%, respectively [94]. Later, another study in Galicia (northwest of Spain) on 401 patients HBV HBsAg positive and HBV DNA positive showed that 259 (64.6%) patients were infected with HBV genotype D, representing 4 subgenotypes: the subgenotype D4 was the most prevalent (59%), followed by D2 (30%), D3 (7%), and D1 (4%), which were mostly represented by young women [95].

HCC prevalence was not well studied in Spain. Thus, in 1999, the first case of HCC in an HIV-infected patient in the participant hospitals was reported. After that, cases of HCC were diagnosed in HIV and HIV/HCV coinfected infected patients. Thus, in 2003, the incidence density rate of HCC in Spain was between 0 and 0.6 cases per 1000 persons/years and increased in 2008 and 2009 to 2.8 cases per 1000 people/year [96]. A recent analysis of the current situation of cases of HCC in Spain was conducted from 1st October 2014 to 31st January 2015 in 73 centers on a total of 720 patients; 686 of which had HCC. Males represented 82%, and the mean age was 67 years. The incidence was estimated at 10-12/100,000 inhabitants, where cirrhosis was detected in 87% of cases. In this study, the main etiologies were alcohol 35%, HCV 30%, alcohol and HCV 15%, and nonalcoholic fatty liver disease 6% [97].

### 1.15. Italy

Prior to 1980, the prevalence of HBsAg chronic carriers in the general population was above 3% in Italy [98, 99] with an increasing gradient from northern to southern Italy where the rate was closer to 5% [53, 100]. In this regard, Italy was among the first countries to propose HBV vaccine to healthcare workers and high-risk populations, 1981 [101]. In the country, the epidemiology of HBV has largely spread over the last 50 years, with a substantial, progressive reduction in the endemicity levels. The main reasons for this change are due to the improvement in socioeconomic conditions associated with a better standard of hygiene, a reduction in family size, the educational and media campaigns against human immunodeficiency virus (HIV) infection, and, finally, the mass vaccination campaign against HBV started in 1991 for all newborn babies and all 12-year-old children [102]. To test HBsAg in blood donors, a one-year study from April 2004 to March 2005 was performed on 31,190 Italian volunteer blood donors from age classes not subjected to universal HBV vaccination of which 100 (0.32%) were positive for both HBsAg and anti-HBc and two for HBsAg (0.01%) alone, suggesting a prevalence rate for HBV infection of 0.33% [103]. More recently, the rate of HBsAg-positive subjects in the open population ranged between 0.8% and 1% [104]. In Italy, prevalence of HBV infection in HD patients ranges from 0.6 to 2.2%, despite the decline of HBV infection worldwide in the general population, according to data from Regional Italian Registries. From January 2016 to January 2017, the prevalence of HBV infection in HD patients was screened by analyzing the HBV serological markers of 322 HD patients in Palermo, Italy, and found 6 HBsAg-positive patients, corresponding to a prevalence rate of 1.8% [105]. About prisoners in Italy, in 2013, a multicenter study on 2265 subjects from several Italian prisons who were tested for HBV showed that 4.4% were HBsAg positive [106].

In Italy, the vast majority (95%) of cases of AHB were characterized by HBV genotype D [107]. However, as Italy experienced unprecedented immigration rates, predominantly from Sub-Saharan Africa and Eastern Europe, other HBV genotypes have been introduced and have been responsible for about 40% of AHB cases [107]. In 2006, a study reported that about 90% of cases with chronic HBV infection were genotype D [108]. Later in 2012, out of the 34 samples from acute hepatitis B patients, 53% were infected with genotype D, 44% with genotype A, and 3% with genotype E. Those patients were Sicilian, and the mode of transmission was primarily sexual. The molecular analysis of the isolates showed that the HBV strains are related to D2 suggesting that this change in the local epidemiology of the HBV infection may be correlated to the immigration [109]. In 2015, genotypes D, A, and F were detected in 49%, 45%, and 6% of 103 patients with AHB, respectively. Such findings reflected the introduction of new genotypes from other countries, and the most frequently risk factor was the unsafe sexual exposure, significantly associated with non-D genotypes [110].

The age-standardized incidence of HCC for the year 2002 in Italy was reported by the GLOBOCAN 2002 database as 15.9/100,000 for men and 5.1/100,000 for women [111].

Another study reported that among 9997 subjects with chronic liver disease (CLD) recruited in 2001 and 2408 recruited in 2014, 3.3% and 5.7%, respectively, had HCC. They also mentioned that the proportion of HBV-related HCC cases showed a decreased rate, reflecting the reduced endemicity of the infection in the country [112].

### 1.16. Turkey

Turkey was placed in the intermediate zone of HBV prevalence. But, over time, we assisted to a decrease in the HBV incidence from 8.26 per 100,000 people in 2002 to 4.26 per 100,000 people in 2010, explained by HBV vaccination to newborn since 1998 [113]. Between January 2007 and December 2009, a retrospective case-control study on 129 HD patients found HBV in 30.2% of cases. For this study, all adult patients and controls were volunteers.

Nonadult patients were included only with their parents' permission [114]. Later in 2015, HBsAg was found in 4% of 5460 participants from both the rural and urban areas with a higher prevalence in the eastern regions. 50.9% of HBsAg-positive subjects were females and the mean age was 40.8 ± 14.7 years [115]. Interestingly, screening of drug abusers between January 2013 and December 2017 demonstrated the existence of HBsAg in 94 out of 4357 patients (2.2%), aging between 14 and 71 years [116]. The prevalence of HBV genotypes in Turkey was screened from February 2001 to February 2002 on 158 patients (99 males and 59 females). Genotype D was found in 147 cases (93.04%): subtype D2 found in 122 cases (94.6%), subtype D1 in five (3.9%) and D2+deletion in two (1.5%) cases [117]. In the same period, between March 2001 and October 2002, a prospective study included 88 patients with CHB infection, aging 16 to 65 years. Genotype D was detected in 88.7% of cases; however, genotyping failed in two patients (2.3%), while no product was obtained in eight (9.0%) patients. Regarding subtypes, D2 was more prevalent (78%-85.9%) followed by subtype D2+deletion (8.9%), subtype D1 (3.9%), and subtype D3 (1.3%) [118]. On the other hand, between June 2002 and February 2003, genotype D was the only type detected in 63 randomly selected CHB patients [119]. The same genotype was detected in 99.1% of 5533 screened volunteers living in urban and rural areas of 23 cities of Turkey during 2009 and 2010 [115]. HCC incidence in Turkey, according to 2003 data from the Ministry of Health, is 0.83/100,000 people/year. In order to determine the epidemiological characteristics, etiological causes, tumor characteristics, and levels of HCC in the country, a study was carried out in 2014 on a total of 963 patients diagnosed with HCC. 758 (79%) were males with a mean age 61.42 ± 11.1 years and 205 (21%) females with a mean age of 60.05 ± 13.8 years. HBV was positive in 555 patients (57.6%) with a higher rate observed in men 81% vs. 19% in women [120]. In Turkey, HCV contributes, mostly, in HCC development in 49% of cases, whereas HBV, alcohol, and other factors are responsible for 26, 19, and 11%, respectively [28].

### 1.17. Greece

Until the early 1980s in Greece, hepatitis B had long been a serious public health problem. After that, thanks to many factors such as demographic and socioeconomic changes, medical precautions, and vaccination introduced in 1998 concerning all newborns as well as adolescents (11 years old), the incidence of the disease decreased [121]. General population published data are limited in Greece, whereas blood donors are the best-studied group. Between 1991 and 1996, the larger cohort of voluntary blood donors formed by military recruits (*n* = 80,302), enlisted military personnel (*n* = 86,920), and directed family donors (*n* = 75,403) was studied in Athens. Results showed the mean prevalence of HBsAg to be 0.84% [122]. From September 1997 to February 1998, 1050 males from Greek Navy recruits, aged between 18 and 25 years, were included in a study. Serological evidence for HBV infection was found in 24 persons (2.28%) of the 1050 examined and in 3.39% of the nonvaccinated population tested. The HBsAg carrier rate was 0.95% and 1.4%, respectively [121]. In 2002, a study was carried out on the general population of Greece on 1500 individuals (females: 52.3% and males: 47.7%) and allowed the detection of differences ranging from 1.2 to 4.8% for HBsAg.

In that period, the prevalence of HBV infection seemed to decrease with a 22.6% rate of HBV markers and a 2.1% rate of chronic HBV carriers [123]. Thus, a study between August 2003 and August 2004 was established on a total of 13,581 women aged between 16 and 45 years. The origins of the study population were diverse: 70.31% were from Greece, whereas 15.96% of them were of Albanian origin, and the rest came from different countries (Eastern European countries, African countries, Asian countries, Northwestern European countries, Australia, and North American countries). Among 13,581 females, only 157 (1.16%) were HBsAg positive and most of these subjects were Albanian (71.34%). These results suggest that the seroprevalence of HBsAg was 5.1% in Albanian women and only 0.29% in Greek ones [124]. According to the recent data, it can be concluded that while Greece is similar to other Mediterranean countries in that it has intermediate prevalence, it is unique in that it has multiple ethnic groups present: this may also explain why women are more affected, as opposed to the usual male dominant pattern. In 2007, genotype D was reported in 100% of the HBV cases in Greece [125]. In 2011, a retrospective study on 135 serum samples (93 males and 42 females) revealed that genotype D was found in 98%, genotype A in 1%, and genotypes B and C in 0.5% for each, which were exclusively found in Asian immigrants [126].

To analyze HCC situation in the country, a geoepidemiological study in Crete Island on a total of 812 cases of cirrhosis (69.1% were males aging between 33 and 86 years) and 321 cases of HCC (73.0% were males aging between 46 and 89 years) was carried out from 1990 to 2014. Crete Island has a genetically homogeneous population and serves to study HCC situation in the country as it regroups more than 600,000 inhabitants. The median age of HCC cases was 70 years and 73% were males. The study reported that the incidence increased from 6 new cases per 100,000 of population in the first five-year period to 16.8 in the last five years. HBV-related cases were relatively constant (1990-1994: 8.3%, 1995-1999: 20.4%, 2000-2004: 13.9%, 2005-2009: 12.1%, 2010-2014: 7.3%) [127].

### 1.18. Croatia

In Croatia, vaccination of high-risk groups has started since 1992. Later in 1999, vaccination concerned only adolescents of 12 years or older. In 2004, mandatory vaccination included the family members of HBsAg-positive people and persons with leukemia. Finally in 2007, obligatory immunization was required for adolescents and children in the first year of life in order to protect the future generations [128]. To highlight the epidemiological characteristics of hepatitis B in Croatia, data of medical histories about persons treated for addiction to psychoactive drugs in Croatia showed a downward trend of HBV-positive patients during the period from 2004 to 2011: in 2004, there were 19.2%, 15.5% in 2006, 13.2% in 2008, 10.4% in 2010, and 7.3% in 2011 [129]. In 2012, the country was described to have a low intermediate prevalence of HBsAg carriers constituting 2-4% of the population [130], while Kaic et al. found a low prevalence of chronic HBV infection, estimated to <2% [131]. In 2015, data from the Croatian national institute of public health, the Croatian health service yearbook, and epidemiological news between 1996 and 2012 showed that the incidence of the disease was 3.67/100,000 people/year and that higher rates of the disease were found in the 15–19 and 20–29 age groups. Infection rate in men was 1.4 times more affected than women.

Additionally, HBsAg prevalence in blood donors decreased from 0.65% in 1992 to 0.012% in 2011 [128]. Concerning HBV genotypes, 100 HBsAg-positive patients with detectable HBV DNA were tested in 2008. Genotype D was most frequent with 80% of cases. Genotype A and mixed genotypes were found in 8% and 12% of patients, respectively [132].

Croatia belongs to Southern Europe countries where the incidence rates of liver cancer are 9.5/100,000 (*n* = 14,135 among 74,900 millions) in males and 2.9 (*n* = 6423 among 78,393 millions) in females and the mortality rates are 7.5/100,000 in males and 2.5/100,000 in females, from the GLOBOCAN database in 2012 [133]. The country showed a decline in incidence among women and an increase in mortality in men, according to the Average Annual Percent Change (AAPC) estimated at -2.9 and 2.4, respectively [133].

## 2. Discussion

In this review, we reported that HBsAg prevalence varies between low and intermediate in all Mediterranean countries, genotype D predominates, and we concluded that men develop the disease and its complications more than women. Many countries (Algeria Tunisia, Libya, Egypt, Syria, Spain, and Italy) are characterized by significant differences observed between the northern and the southern governorates studied, revealing an important heterogeneity in HBV transmission within the same governorate. In some studies, prevalence in some specific groups (blood donors, sex workers, drug users, and prisoners) was higher than that among the general population. This may be due to the limited number of participants, not reflecting a representative portion of the population. In this review, it has been reported that chronic hepatitis B is much more frequent in men than women, and in all Mediterranean countries, males are more prone to develop HCC than females [11]. This gender difference is probably due to natural protective influences of estrogen against liver inflammation in women, higher prevalence of HBV and higher exposure to carcinogen such as tobacco and alcohol, except for Greece where the HBV infection is more prevalent in women than men. In addition, HCC was higher in men than women in all reported countries. To confirm the HCC male-to-female ratio, an analysis on a total of 44,287 incident cases of HCC (33,196 males and 11,091 females) was carried out in the United States during the period 1992-2013 on different ethnicities (blacks, Asian and Pacific Islanders, non-Hispanic whites, Hispanic whites, and American Indian/Alaska native) and revealed an overall male-to-female ratio in age-standardized rate of 3.55. In the study, the male/female ratio decreased after the ages 45-49 years or older; in all racial/ethnic groups [134]. This may be explained by the decreased hormone levels in females after the menopausal ages. In addition, in Europe, infection with hepatitis viruses has less of a responsibility, as excessive alcohol consumption comes first [85]. In contrary, in Arabic countries, alcohol abuse accounts only for about 10% to 19% in Turkey and from 15 to 20% in Lebanon [28, 135]. The Maghreb region is also characterized by a low consumption of alcohol, estimated at 17.9% of HCC patients, an intermediate endemicity for chronic hepatitis B, and a low rate of HCV carriage (except in Egypt). Thus, in that region (Maghreb), HBV contributes in only 17.9% of cases in HCC development [135], whereas HBV infection remains the most important risk factor of HCC in other parts of the Near- or Middle-East (Saudi Arabia, Lebanon, Iran, and Turkey). In Greece, authors attribute the raise of HCC incidence in the country to a gradual increase in the incidence of HBV and alcohol-related cases, especially during the last decade [127]. However, development of HBV vaccine has been a major success in reducing the incidence of HBV infection and subsequent development of HCC. The vaccine effectiveness is remarkable, whether alone or combined. In children, its effectiveness exceeds 90% and, comparing the era before vaccination and 2015, the WHO reported that global coverage with the three doses of hepatitis B vaccine in infancy reached 84% in 2015 and resulted in a decrease in the global proportion of children under 5 from 4.7% to 1.3% [136]. Moreover, the prevalence of HBsAg was markedly reduced and less than 1% of the population of Western Europe and North America is chronically infected [104]. Besides, the complete vaccination confers a protection of 20 years, possibly even for life [127]. To test the persistence of long-term immunogenicity against HBV among adolescent people, a cohort of 520 nursing students two decades after primary vaccination was carried out at the University of Palermo. The students were evaluated for levels of anti-HBsAg antibodies showing a difference between receiving HBV vaccination at adolescence and at infancy. Vaccinated students at adolescence showed a significant association with an increased possibility of having anti-HBsAg titers ≥ 10 mIU/mL than those who were vaccinated at infancy (69% vs. 31.7%) [137]. But, on the other hand, the vaccine is not effective against an already established infection nor against escape mutants at vaccination. Also, age, alcohol, tobacco, obesity, or people with failures in the immune system are factors limiting the response to the vaccine [138]. Moreover, in some countries, despite the introduction and high coverage of universal vaccination among preadolescents, HBV continues to be transmitted among unvaccinated older children, adults, and immigrants after war or from countries with high or intermediate prevalence where hepatitis B vaccination programs remain unimplemented or where coverage is low [28].

## 3. Conclusion

In the Mediterranean area, the prevalence of HBsAg is still low to intermediate and the HBV genotype that dominates is genotype D. In addition, most studies confirm that men are more affected by HBV than women. Concerning HCC, it is also more frequent in men than women and it develops in most cases in a context of chronic liver disease and most often on cirrhosis. Finally, according to some countries' results, children must be admitted to the national HBV vaccination schedule, so that hepatitis infections and their complications could be early prevented.

## Figures and Tables

**Table 1:** HBsAg prevalence and date of universal vaccination in the different Mediterranean countries.

	General population (%)	Blood donors (%)	HD patients (%)	Pregnant women (%)	Others (%)	Vaccination date	References
Morocco	1.81	0.8–0.96	6	1.2	ND	1999	[17, 19–22]
Algeria	2.15	3.6	3.2	1.6	ND	2002	[29–32]
Tunisia	4.2–5.6	0.8	5.5	4	ND	1995	[36–39]
Libya	2.2	0.21	2.6	ND	1.1 (health workers)	1993	[45, 47, 49, 50]
Egypt	1.4	1.3	2	4	1.4 (health workers)	1992	[52–57]
Syria	4.2	ND	0.63–2.52	1.1	5.3 (drug users) and 10.8 (sex workers)	1993	[63–65]
Lebanon	1.74	ND	ND	ND	0.99 (sex workers) and 2.4 (prisoners)	1998	[69–71]
Palestine/Israel	1.75	ND	3.8	ND	ND	1992	[44, 74, 75]
France	0.65	0.12	0.84	0.65	ND	1982	[78–81, 83]
Spain	<0.27–1.69	ND	1.03	0.8	0.7 (healthy employed population)	1990	[89–94]
Italy	0.8–1	0.01–0.32	1.8	ND	4.4 (prisoners)	1981	[98, 103–106]
Turkey	4	ND	30.2	ND	2.2 (drug users)	1998	[113–116]
Greece	1.2–4.8	0.84	ND	ND	ND	1998	[121, 122]
Croatia	2–4	0.012	ND	ND	7.3 (drug users)	1992	[128–130]

ND: no data.

**Table 2:** HBV genotypes and subgenotypes in the different Mediterranean countries.

	HBV genotype	HBV subgenotype	References
Morocco	D (97.5%), A (2.5%)		[16]
	D (100%)		[18, 23]
	D (90.45%), A (5.9%), E (0.5%), and mixed (A/D and D/F) (3.17%)	D7 (63.3%), D1 (32.7%), D4 (2%), D5 (2%), and A2	[24]

Algeria	D (93%), A (5%), and E (2%)		[34]
	D (86.5%) and A (11.76%)	D7 (43.5%), D3 (24.75%), D1 (16.8%), D2 (14.85%), and A2 (11.76%)	[35]

Tunisia	D (80%), A (9%), and E (8%)	D7	[40]
	D (84.75%), A (0.6%), B (0.6%), C (1.82%), and 20 mixed genotypes (12.2%)		[41]
	D (96%), A (4%)	D1 (55%), D7 (41%), and D3 (3%)	[42]

Libya	D (90%), A (1.7%), E (1.7%), and D/E (6.7%)	ND	[51]

Egypt	D (100%)		[58]
	D (87%) and mixed D/F (13%)		[59]
	E (50%), D (21.43%), and coinfection D/E (28.57%)	D1 (21.43%)	[57]
	D (37.1%) and B (25.7%)		[61]

Syria	D (97%)	ND	[67]
	D (100%)		[68]

Lebanon	D (100%)	D1 (57.38%) and D2 (11.48%)	[72]

Palestine/Israel	HBV/HDV: D (100%)	D2 (66.7%), D1 (16.7%), and D3 (16.7%)	[75]
	HBV: D (88.9%), A (7.4%), and C (3.7%)	D1 (59.3%), D2 (18.5%), D3 (11.1%), and A1, A2, and C2 (3.7%)	
	D (92.5%) and A (7.5%)	D1 (90%), D3 (2.5%), and A2 (7.5%)	[76]

France	G	ND	[84]
	D (29%), A (24%), C (11%), and E (10%)		[85]
	D (27%), A (24%), E (13%), C (12%), B (7%), and mixed genotypes (16%)		[86]

Spain	A (52%), D (35%), and F (7%)	D4 (59%), D2 (30%), D3 (7%), and D1 (4%)	[94]
	D (64.6%)		[95]

Italy	D (95%)		[107]
	D (90%)		[108]
	D (53%), A (44%), and E (3%)	D2	[109]
	D (49%), A (45%), and F (6%)		[110]

Turkey	D (93.04%)	D2 (94.6%), D1 (3.9%), and D2+deletion (1.5%)	[117]
	D (88.7%)	D2 (78%-85.9%), D2+deletion (8.9%), D1 (3.9%) and (1.3%)	[118]
	D (100%)		[119]
	D (99.1%)		[115]

Greece	D (100%)	ND	[125]
	D (98%), A (1%), B (0.5%), and C (0.5%)		[126]

Croatia	D (80%), A (8%), and mixed genotypes 12%	ND	[132]

ND: no data.

**Table 3:** Incidence and etiologies of HCC in the different Mediterranean countries.

	HCC (people/year)	% male/female	Etiology (%)				References
			HBV	HCV	Alcohol	Other	
Morocco	<4/100,000	Male: 62.5% vs. female: 37.5%	31	36	14	19	[26–28]
Algeria	4-7.9/100,000	ND	ND				[27]
Tunisia	1.49/100,000	Male: 90.33% vs. female: 9.67%	20	44	18	18	[28, 43]
Libya	8-11.9/100,000	ND	33	34	15	18	[27, 28]
Egypt	>20/100,000	81.5% male and 18.5% female	13	63	12	12	[27, 28]
Syria	ND	ND	32	34	14	19	[28]
Lebanon	ND	ND	28	40	17	15	[28]
Palestine/Israel	0.75/10,000	ND	20	48	15	17	[28, 77]
France	29.37/100,000; 5.39/100,000 2.9/100	Male: 88% vs. female: 12% Male: 62% vs. female: 38%	ND	15.51	84.49	ND	[87, 88]
Spain	2.8/1000 10-12/100,000	Male: 82% vs. female: 18%	ND	30	35	6-15	[96, 97]
Italy	Male:15.9/100,000 Female: 5.1/100,000	ND	ND				[111, 112]
Turkey	0.83/100,000	Male: 81% vs. female: 19%	26	44	19	11	[28, 120]
Greece	16.8/100,000	Male: 84% vs. female: 16%	7.3	ND			[127]
Croatia	Male: 9.5/100,000 Female: 2.9/100,000	ND	ND				[133]

ND: no data.
